# Neonatal thrombocytopenia—causes and outcomes following platelet transfusions

**DOI:** 10.1007/s00431-018-3153-7

**Published:** 2018-04-28

**Authors:** Elisabeth Resch, Olesia Hinkas, Berndt Urlesberger, Bernhard Resch

**Affiliations:** 1 Research Unit for Neonatal Infectious Diseases and Epidemiology, Medical University of Graz, Graz, Austria; 2 Division of Neonatology, Department of Pediatrics and Adolescent Medicine, Medical University of Graz, Auenbruggerplatz 34/2, 8036 Graz, Austria

**Keywords:** Neonatal thrombocytopenia, Platelet transfusion, Mortality, Bleeding

## Abstract

We evaluated the causes for neonatal thrombocytopenia (NT), the duration of NT, and the indications of platelet transfusions (PT) by means of a retrospective cohort study over a 23-year period. Neonates with NT were identified via ICD-10 code D69.6. Of 371 neonates (1.8/1000 live births) with NT, the majority (312; 84.1%) had early onset thrombocytopenia, and 282 (76%) were preterm born. The most frequent causes for NT were early and late onset sepsis and asphyxia. The mean duration of thrombocytopenia was 10.2 days and was negatively correlated (KK = − 0.35) with the number of PT. PT were given to 78 (21%) neonates, 38 (49%) of whom had very severe NT. The duration of NT was positively related to the severity of NT and the number of subsequent PT. A mortality rate of 10.8% was significantly associated with bleeding signs (*p* < 0.05) and correlated with increasing number of PT (*p* < 0.05) but not with the severity of NT (*p* = 0.4). In the case of relevant hemorrhage, PT did not influence the mortality rate (*p* = 0.09). All deaths followed neonatal sepsis.

*Conclusions*: Prematurity and diagnoses including early and late onset sepsis and asphyxia were the most common causes of NT. Mortality was not associated with the severity of NT but increased with the number of PT.

**Table:** 

**What is Known:**	
*• The causes for neonatal thrombocytopenia (NT) are well known.*	
*• The effects of platelet transfusions (PT) and its indications are still a matter of debate and recommendations differ widely.*	
**What is New:**	
*• The duration of NT is positively related to the severity of NT and the number of subsequent PT.*	
*• The mortality rate is not associated with the severity of NT but increases with increasing numbers of PT and in the case of relevant intraventricular hemorrhage (≥ grade II), PT does not influence the mortality rate.*

## Introduction

Thrombocytopenia develops in 18–35% of neonates admitted to intensive care units. In addition, Sola et al. [1] showed that the likelihood of developing thrombocytopenia increases with the degree of prematurity. Roberts and Murray [2] calculated that low-birthweight infants were at a 2.52-fold increased risk for thrombocytopenia. The rate and severity of thrombocytopenia in neonates of pregnancy-induced hypertensive mothers vary. Chakravorty and Roberts [3] demonstrated in those pregnancies complicated by preeclampsia that the usual course of thrombocytopenia includes diagnosis within the first 2–3 days and resolution by 7–10 days of life in most cases.

Platelet production, or thrombopoiesis, is a complex process that results in the production of thrombopoietin as the thrombopoietic stimulus leading to the generation and proliferation of megakaryocyte progenitors.

Platelet transfusions (PT) in neonatal thrombocytopenia (NT) are commonly administered to reduce the risk of bleeding. However, there are few evidence-based guidelines to inform clinicians’ decision-making processes. Developmental differences in hemostasis and differences in underlying disease processes make it difficult to apply PT practices from other patient populations to neonates [4]. Specifically, it is important to identify neonates at risk of bleeding who would benefit from PT and to determine whether PT either abrogate or exacerbate common neonatal complications such as sepsis, chronic lung disease, necrotizing enterocolitis (NEC), and retinopathy of prematurity. Among 972 very-low-birthweight (VLBW) infants from a multicenter retrospective cohort study, Sparger et al. [5] reported that 231 (24%) had received a total of 1002 PT. A large proportion of PT were given to VLBW infants with platelet counts greater than 50,000/μL. Additionally, they found that the severity of illness influenced transfusion decisions. However, the severity of NT did not correlate with the risk of intraventricular hemorrhage (IVH), and in contrast, PT did not reduce the risk of IVH [5].

The aim of the study was to evaluate the causes for NT, the duration of NT, and the indications and effects of PT by means of a retrospective cohort study.

## Methods

All infants with a diagnosis of NT born between January 1, 1990 and December 31, 2012 at the Division of Neonatology of the Department of Pediatrics of the Medical University Graz, a tertiary care center in the southern part of Austria, were included retrospectively for analysis. The study was approved by the ethics committee of the Medical University of Graz (number 26-249 ex 13/14). We searched for NT using ICD-10 code D69.6 and data from our local electronic database of the Division of Neonatology.

Maternal (age; number of pregnancies; maternal diseases including idiopathic thrombocytopenic purpura, systemic lupus erythematosus, and malignancies; maternal medication including nonsteroidal anti-inflammatory drugs, chignolin alkaloids, and heparin; and pregnancy-induced hypertensive disorders), perinatal (multiple birth; in-vitro fertilization; gestational age in weeks; birth weight in grams; small for gestational age; intrauterine growth restriction; placental vascular abnormalities including infarction, Apgar score at 1, 5, and 10 min, and umbilical artery pH), and neonatal data (gender, early and late onset sepsis (EOS/LOS), necrotizing enterocolitis (NEC), intra-periventricular hemorrhages [I/PVH], TORCH infections, asphyxia, neonatal alloimmune thrombocytopenia, hemolytic disease of the newborn, myeloproliferative disorders, chromosomal disorders, metabolic diseases, and mortality) were collected. IVH grade ≥ II was defined as relevant intracranial hemorrhage.

NT was defined as a platelet count less than 150,000/μL [6] based upon the definition used in adults, which corresponds to values at or below the fifth percentile. The degree of thrombocytopenia was defined as follows: mild, a platelet count of 100,000 to 150,000/μL; moderate, a platelet count of 50,000 to < 100,000/μL; severe, a platelet count of 30,000 to < 50,000/μL; and very severe, a platelet count of < 30,000/μL [6]. Neonatal thrombocytopenia has been categorized into two groups depending on the time of onset: early onset (EOT), which is within 72 h of life and late onset (LOT), after 72 h of life [7, 8]. The duration of NT was defined as the time from diagnosis to normalization (values above 150,000/μL). Normalization of NT with PT required at least values above 150,000/μL 48 h apart. Our own policy was to give PT in preterm infants or with signs of hemorrhage at counts below 50, 000/μL and in every case of counts below 30, 000/μL.

Asphyxia was defined according to the Statement of the American Academy of Pediatrics and the American College of Obstetricians and Gynecologists: umbilical artery pH ≤ 7.0 and base deficit ≥ 12 mmol/L, clinical signs of encephalopathy, and/or Apgar score at 5 min 0–3 [9]. I/PVH were classified according to the description of Papile et al. [10]. Other bleeding conditions included pulmonary hemorrhage; gastrointestinal bleeding; cutaneous bleeding (petechial, ecchymosis, and hematoma); umbilical bleeding; and hematuria.

Statistical analysis was carried out using Open Office calc 4.0. The *t* test and Wilcoxon test were used for numerical data, and the chi square test using Yates correction and Fisher’s exact test, as appropriate, were employed for categorical data. For all statistical tests, a level of significance of 0.05 was used. Correlations with maternal, neonatal, or perinatal data were performed using Pearson’s correlation coefficient.

## Results

The study sample comprised 371 neonates, 312 (84.1%) of whom had EOT and 59 (15.9%) LOT. The majority had mild (*n* = 122, 33%) to moderate (*n* = 139, 38%) NT; only 14% (*n* = 52) had a severe NT and 15.6% (*n* = 58) a very severe NT.

The median gestational age of the study population was 33 (range = 23–42) weeks. The majority (76%) were born preterm: 76% in the case of EOT and 77% in the case of LOT. The percentage of preterm infants with a gestational age of < 28 weeks was 20 and 39% in the case of EOT and LOT, respectively (*p* = 0.09). Prematurity was not associated with the severity of NT (*p* = 0.14). The prevalence of NT in preterm infants was 4% (282/6964) during the study period. With approximately 200,000 live births in our region (Southern Styria) over the study period, we calculated the incidence of NT as 1.8/1000 live births.

Perinatal data are given in Table 1.

**Table 1:** Perinatal data on 371 neonates with neonatal thrombocytopenia

Perinatal Data	Numbers
Maternal age (years)	30 (15–45)
Number of pregnancies	2 (1–10)
Multiple pregnancy	65 (17.5)
Intrauterine growth restriction	99 (26.7)
Cesarean section	280 (75.5)
Breech presentation	74 (20)
Gestational age (weeks)	33 (23–42)
Birth weight (grams)	1760 (383–5300)
Gender (male/female)	215:156 (58:42)
Small for gestational age	111 (30)
Apgar score after 1 min	7 (0–9)
Apgar score after 5 min	9 (0–10)
Umbilical artery pH	7.26 (6.38–7.94)

Data are given as *n* (%) or median (range)

Maternal morbidities found associated with NT included preeclampsia (*n* = 35, 9.4%); HELLP syndrome (*n* = 13, 3.5%); immune thrombocytopenia (*n* = 12, 3.2%); pregnancy-induced hypertension (*n* = 10, 2.7%); systematic lupus erythematosus (*n* = 4, 1.1%); and cancer (*n* = 1, 0.27%). Maternal drug history possibly associated with NT included heparin (*n* = 7, 1.9%), nonsteroidal anti-inflammatory drugs (*n* = 3, 0.8%), and quinolone alkaloids (*n* = 1, 0.27%).

Neonatal diagnoses found associated with NT are listed in Table 2. Sepsis was the most common morbidity associated with NT; 122 cases (40.2%) were associated with EOT and 52 (74.2%) with LOT (*p* < 0.001).

**Table 2:** Neonatal diagnoses found in thrombocytopenia of 371 neonates

Neonatal diagnoses	Number (%)
Sepsis	175 (47)
Early onset sepsis	128 (73)
Late onset sepsis	47 (27)
Asphyxia	95 (25)
Necrotizing enterocolitis (Bell stage ≥ IIa)	16 (4.1)
Chromosomal anomalies	15 (3.9)
Hemolytic disease of the neonate	9 (2.4)
Cytomegalovirus infection	9 (2.4)
Myeloproliferative disease	6 (1.6)
Neonatal alloimmune thrombocytopenia (NAIT)	4 (1.0)
Kasabach–Merritt syndrome	2 (0.5)
Metabolic disorders	2 (0.5)
Thrombosis	2 (0.5)

Severe and very severe NT were found in cases of sepsis (both EOS and LOS), NEC, asphyxia, chromosomal anomalies, Kasabach–Meritt syndrome, hemolytic disease of the neonate (HDN), NAITP, myeloproliferative disease, cytomegalovirus infection, and thrombosis. Transient NT of unknown origin was always mild. Severe and very severe NT were significantly associated with sepsis and predominantly with LOS (*p* = 0.003). A mild or moderately severe NT tended to occur more often in case of EOT (*p* = 0.067).

Hemorrhage was detected in 188 (50.7%) cases. They are listed in Table 3. I/PVH occurred in 104 cases (28%) and IVH grade ≥ II in 68 (18.3%). There was no association between the severity of NT and I/PVH in contrast to cutaneous bleedings (*p* = 0.006). No other hemorrhages were associated with the severity of NT.

**Table 3:** Bleeding conditions in 371 neonates with neonatal thrombocytopenia

Bleeding condition	Number (%)
Intra-/periventricular hemorrhage (I/PVH)	104 (55.3)
IVH I	36 (19.2)
IVH II	15 (8.0)
IVH III	26 (13.8)
PVH	27 (14.3)
Cutaneous bleeding	46 (24.5)
Gastrointestinal bleeding	16 (8.5)
Pulmonary hemorrhage	14 (7.4)
Hematuria	4 (2.1)
Umbilical cord bleeding	3 (1.6)
Adrenal gland hemorrhage	1 (0.6)

PT were given in 78 neonates (21%). Thrombocyte counts were below 30,000/μL in 38 neonates (48.8%); 16 neonates (20.5%) had severe, 21 (26.9%) had moderate, and 3 (3.8%) had mild NT with signs of bleeding. A single PT was given in 50 cases (64%), leading to subsequent normalization of thrombocyte counts, and in 22 cases (28.2%), two or more PT were necessary. Data were not available for 10 cases (7.8%).

The duration of NT could be calculated for 288 cases. The mean duration was 10.2 days: 8.9 days for EOT and 16.8 days for LOT (*p* < 0.001). The mean duration of NT was 8.5 days without PT and 18.3 days with PT (*p* < 0.001). The duration of NT was positively related to its severity (mean duration = 4–21 days). The duration of NT correlated negatively with the number of PT (KK = − 0.35). Following one PT, the mean duration of NT was 16.6 days, and following multiple PT, it was 28 days. PT did not shorten the duration of NT.

In the study group, 40 (10.8%) neonates died; details are provided in Table 4; 36 (90%) had EOT, and 4 (10%) had LOT, and 11 (27.5%) died within 7 days of life. At least 30 (75%) neonates were still thrombocytopenic at death. The mortality rate of neonates was 12.5% (*n* = 23) with and 9.1% (*n* = 17) without bleeding conditions (*p* < 0.05). Thus, mortality was significantly associated with bleeding signs (*p* < 0.05) and correlated with increasing number of PT (*p* < 0.05). The mortality rate was not associated with the severity of NT (*p* = 0.4). In the case of severe I/PVH, PT did not influence the mortality rate (*p* = 0.09).

**Table 4:** Single center analysis of 40 deaths having had neonatal thrombocytopenia between 1990 and 2012

Reason of death	Number	Percent (%)
Septic shock and multiorgan failure	8	20
Postasphyxial multiorgan failure	7	17.5
Intra-/periventricular hemorrhage	7	17.5
Necrotizing enterocolitis	2	5
Twin-to-twin transfusion syndrome	2	5
Trisomy 18	2	5
Congenital cytomegalovirus infection	1	2.5
Hemolytic disease of the neonate (Kell)	1	2.5
Hemophagocytic lymphohisticytosis	1	2.5
Autoimmune proliferative syndrome	1	2.5
Neuroblastoma grade IV	1	2.5
Congenital diaphragmatic hernia on ECMO	1	2.5
Autosomal recessive polycystic kidney disease	1	2.5
Cardiac shock (supraventricular tachycardia)	1	2.5
Congenital leukemia	1	2.5
Neonatal alloimmune thrombocytopenia	1	2.5
Unknown	2	5
Parameter of dead neonates	Median	Range
Age at death (days)	5	1–95
Gestational age (weeks)	28	23–41
Birth weight (grams)	1015	383–4620
	Number	Percentage
Male gender	21	52.5
Preterm born	32	80
Extremely low gestational age (≤28 weeks)	23	57.5

## Discussion

Our study demonstrated that prematurity, sepsis, and asphyxia were the most common factors associated with NT. Our mortality rate of 10.8% was comparable to the 7–10% rate reported in the literature [11, 12]. Mortality was not associated with the severity of NT but increased with the number of PT.

The majority of neonates had mild to moderate NT (71%) and only 15% had very severe NT. Von Lindern et al. [11] reported a 68% rate of mild to moderate NT and the same rate for very severe NT (16%). The severity of NT was not associated with gender, gestational age, birth weight, small for gestational age (SGA), or breech presentation, as reported elsewhere [11].

Three quarters of neonates were preterm neonates. The association between NT and prematurity or low birth weight is well documented [4, 7, 11, 13–15]. In this context, SGA is a well-known risk factor for developing NT [11, 16, 17] and rates have been reported as high as 41 and 53%, respectively [16, 18]. Thus, our rate of 30% was not surprising. The finding of a prolonged course of NT in LOT is well-documented [17, 18].

### NT-associated findings

Our sepsis rate associated with NT was higher than reported by Ulusoy et al. [7] and von Lindern et al. [11]: 47% compared with 30 and 36%, respectively. The finding that severe and very severe NT were significantly associated with sepsis was again confirmed by von Lindern et al. [11]. Regarding asphyxia, we found lower rates in the literature (3–11%) [7, 11]. NEC is a morbidity often complicated by NT. Our rate of 4.1% was twice as high as reported elsewhere [7, 11]. Other associations, including chromosomal anomalies, HDN, metabolic disorders, and thromboses, ranged between 0.5 and 3% [7, 11], except the 10% rate of HDN reported by von Lindern et al. [11].

Our NT rate of 4% in preterm infants is fairly low compared to a 12% rate reported by Kusamari et al. [19]. The majority of studies report higher rates of NT ranging between 22 and 35% [1, 2, 11, 13, 20, 21]. Astonishingly high rates (53–70%) have been reported from developing countries [22, 23].

### NT and bleeding conditions

The prevalence of hemorrhage in thrombocytopenic neonates was approximately 20–30% according to the literature and thus markedly lower than our rate of 50% [14, 24]. The risk of hemorrhage was found to be associated with lower gestational age, definite causes of thrombocytopenia, and the severity of concomitant morbidities [11, 17, 25].

Severe sepsis and NEC have been identified as the most common diagnoses in bleeding neonates in an observational study including 169 neonates with severe NT [12]. In those neonates with mild or no hemorrhage, the most common cause of severe NT was intrauterine growth restriction and maternal pregnancy-induced hypertension [12].

A causal link between thrombocytopenia and IVH has not been established, and PT could not reduce the risk of IVH as shown by our data [4]. According to some studies, most preterm neonates who have developed major IVH become thrombocytopenic during the subsequent course of bleeding, as opposed to thrombocytopenia being the cause of IVH [26, 27]. Additionally, considering IVH as a multifactorial event, it seems highly unlikely that an isolated low platelet count leads to bleeding [20].

Interestingly, comparable rates of IVH have been reported unrelated to the severity of NT [14]. In contrast, cutaneous bleeding conditions were significantly associated with the severity of NT [26], as shown by our data. The prevalence of skin bleeding in thrombocytopenic neonates has been reported as up to 81% [28].

### Indications for PT

PT remains a common therapeutic measure in the treatment of severe NT. Generally accepted cutoff values are missing, and PT is a costly therapy involving potential risks such as non-hemolytic febrile reactions, allergic reactions, PT-associated lung insufficiency, and infections [29, 30]. In contrast to other age groups, a higher number of adverse events has been reported in neonates [31]. Therefore, indications for PT should be based on commonly accepted criteria and the best therapy might be correction of the underlying disorder. Most studies report an increased risk of bleeding at thrombocyte counts below 20,000/μL [32–34]. Our results show a wide range of thrombocyte counts associated with PT, reflecting uncertainty among clinicians and a lack of data on evidence-based transfusion criteria [4].

### Recommendations for PT

Many studies and reviews deal with the optimal criteria for platelet transfusion in cases of NT [3, 20, 32, 35, 36]. An overview of current recommendations is given in Table 5 [2, 3, 20, 21, 32, 37–44]. Most authors recommend PT at the cutoff value of 50 × 10^9^/L in case of active neonatal bleeding, and many authors recommend PT in case of prematurity alone. Prophylactic PT are definitely recommended at values below 20 × 10^9^/L and probably recommended at values below 30 × 10^9^/L. Otherwise, the neonatologist is advised to take into consideration possible risks associated with PT outweighing the severity of associated morbidity. As Sola-Visner and Bercovitz stated, platelet counts frequently drive the decision of whether or not to transfuse, despite the existence of little evidence of what a safe platelet nadir is in both full-term and preterm neonates [4]. According to Baer et al. [26], PT should not be given to neonates with a very low risk for IVH (i.e., weight > 1500 g and/or age > 7 days) solely to maintain the platelet count above arbitrary levels; the only indication might be bruising or bleeding. Most PT are still given prophylactically and not in cases of severe thrombocytopenia and/or bleeding [20, 32, 45, 46].

**Table 5:** Overview of published recommendations for treatment of neonatal thrombocytopenia with platelet transfusions [2, 3, 12, 20, 21, 32, 37–44]

Platelet count	Guideline	Author
< 20 × 10^9^/L	Non-bleeding stable term	Blanchette et al. [38, 39]
	Non-bleeding stable neonate	Gibson et al. [42]
	All neonates (prophylactic)	Chakravorty and Roberts [3]; Carr et al. [43]
20–29 × 10^9^/L	Non-bleeding stable preterm and non-bleeding sick term	Blanchette et al. [38]
	Non-bleeding term	Roberts and Murray [39]
	Non-bleeding stable neonates	Calhoun et al. [40]; Sola-Visner et al. [21]; Murray [32]; Gibson et al. [42]
	Bleeding neonate	Murray et al. [32]; Roberts and Murray [2]
	Non-bleeding neonate and neonate with major bleeding	Roberts and Murray [12]
	Clinically unstable neonate, < 1000 g and < 1 week of age, previous major bleeding (grade 3–4 IVH), current minor bleeding, coagulopathy, requiring surgery or exchange transfusions	Chakravorty and Roberts [3]
	Stable preterm	Carr et al. [43]
	All neonates	Sparger et al. [44]
30–49 × 10^9^/L	Non-bleeding stable preterm and active bleeding neonate (failure of platelet production)	Blanchette et al. [37]
	Active bleeding neonate in all cases	Blanchette et al. [38]
	Non-bleeding sick preterm	Blanchette et al. [38]; Calhoun et al. [40]; Murray [41]
	Non-bleeding stable preterm and non-bleeding sick preterm with DIC and active bleeding neonate (in the case of minor bleeding)	Roberts and Murray [39]
	Active bleeding neonate	Roberts and Murray [2]; Gibson et al. [42]
	Clinically unstable neonate, neonates < 1000 g and ≤ 7 days, previous major hemorrhage (grade 3–4 IVH or pulmonary hemorrhage), current minor bleeding, concurrent coagulopathy, requires surgery or exchange transfusion	Roberts and Murray [2, 12]
	Clinically unstable neonate, neonates < 1500 g during the first week of life, neonate with concurrent coagulopathy, before and after invasive procedures	Sola-Visner et al. [21]
	Major hemorrhage	Chakravorty and Roberts [3]
	Clinically unstable neonate, neonates < 1500 g and ≤ 7 days, concurrent coagulopathy, previous significant hemorrhage (grade 3–4 IVH), prior to surgical period, post-operative 72 h	Sparger et al. [44]
50–99 × 10^9^/L	Non-bleeding sick preterm and active bleeding neonate (in case of DIC)	Blanchette et al. [37]
	Active bleeding neonate (in case of DIC)	Blanchette et al. [38]
	Non-bleeding sick preterm (if platelets fall rapidly) and active bleeding neonate (in case of major organ bleeding)	Roberts and Murray [39]
	Active bleeding neonate	Murray [41]; Murray et al. [32]; Roberts and Murray [2]; Sola-Visner et al. [21]
	Neonate with major bleeding	Roberts and Murray [12]
	Active bleeding neonate, NAIT with intracranial bleeding, before or after neurosurgical procedures	Sparger et al. [44]

*DIC* disseminated intravascular coagulation, *NAIT* neonatal alloimmune thrombocytopenia, *IVH* intraventricular hemorrhage, *Neonate(s)* preterm and term infants if not otherwise specified

### NT and mortality

The association of increased mortality rates with an increasing number of PT mainly reflects the severity of the underlying disease or extreme prematurity. In our study, three quarters of the neonates were preterm infants. Those born below 28-week gestational age are at especially increased risk for I/PVH. Additionally, a correlation of severity of thrombocytopenia with an increased risk of mortality has been described, and preclinical data suggest that thrombocytopenia contributes to mortality rather than simply being representative of disease severity [4]. Three studies from the USA, the UK, and Mexico reported higher mortality rates in neonates who had received PT compared with those who had not [32, 45, 46]. The direct effects of PT appear to be questionable, as specific effects have not been properly evaluated, and the influence of preexisting morbidity is difficult to evaluate [12]. Regarding NEC and NT, Kenton et al. [47] did not find an improvement in mortality with an increasing number or volume of PT.

Some limitations have to be mentioned that mainly include the retrospective design of the study and the single-center analysis that might have influenced completeness of data collection and data lost to follow-up. Nevertheless, data acquisition was carefully done reporting on a large cohort. Despite the long study time period, management did not change over time regarding definitions of NT and management of NT inclusive PT.

In conclusion, prematurity and diagnoses including sepsis, NEC, and asphyxia were the most common causes of NT. Mortality was not associated with the severity of NT but increased with the number of PT. There is still room for improvement regarding safe and evidence-based recommendations for PT, and future strategies including treatment with thrombopoietic agents might be promising for the non-bleeding neonate.

## Abbreviations

EOS: Early onset sepsis
EOT: Early onset thrombocytopenia
IVH: Intraventricular hemorrhage
LOS: Late onset sepsis
LOT: Late onset thrombocytopenia
NEC: Necrotizing enterocolitis
NT: Neonatal thrombocytopenia
PT: Platelet transfusions
